# Reliability and validity of the Spanish version of the 10-item Connor-Davidson Resilience Scale (10-item CD-RISC) in young adults

**DOI:** 10.1186/1477-7525-9-63

**Published:** 2011-08-05

**Authors:** Blanca Notario-Pacheco, Montserrat Solera-Martínez, María D Serrano-Parra, Raquel Bartolomé-Gutiérrez, Javier García-Campayo, Vicente Martínez-Vizcaíno

**Affiliations:** 1 Social and Health Care Research Center, University of Castilla-La Mancha, Cuenca, Spain; 2 Faculty of Nursing, University of Castilla-La Mancha, Cuenca, Spain; 3 Faculty of Nursing, University of Castilla-La Mancha, Albacete, Spain; 4 Miguel Servet Hospital. University of Zaragoza. Aragon's Institute of Health Sciences, Spain

**Keywords:** Resilience, 10-item CD-RISC, Young adults, Reliability, Validity, Questionnaire

## Abstract

**Background:**

The 10-item Connor-Davidson Resilience Scale (10-item CD-RISC) is an instrument for measuring resilience that has shown good psychometric properties in its original version in English. The aim of this study was to evaluate the validity and reliability of the Spanish version of the 10-item CD-RISC in young adults and to verify whether it is structured in a single dimension as in the original English version.

**Findings:**

Cross-sectional observational study including 681 university students ranging in age from 18 to 30 years. The number of latent factors in the 10 items of the scale was analyzed by exploratory factor analysis. Confirmatory factor analysis was used to verify whether a single factor underlies the 10 items of the scale as in the original version in English. The convergent validity was analyzed by testing whether the mean of the scores of the mental component of SF-12 (MCS) and the quality of sleep as measured with the Pittsburgh Sleep Index (PSQI) were higher in subjects with better levels of resilience. The internal consistency of the 10-item CD-RISC was estimated using the Cronbach α test and test-retest reliability was estimated with the intraclass correlation coefficient.

The Cronbach α coefficient was 0.85 and the test-retest intraclass correlation coefficient was 0.71. The mean MCS score and the level of quality of sleep in both men and women were significantly worse in subjects with lower resilience scores.

**Conclusions:**

The Spanish version of the 10-item CD-RISC showed good psychometric properties in young adults and thus can be used as a reliable and valid instrument for measuring resilience. Our study confirmed that a single factor underlies the resilience construct, as was the case of the original scale in English.

## Background

Resilience has been defined as a protective factor against mental problems and as a dynamic process of adaptation to changes in life circumstances [1,2]. Various instruments are available for measuring resilience [3,4]. The Connor-Davidson Resilience Scale (CD-RISC) [5] is a self-administered scale of 25 items that exhibits excellent psychometric properties in young adults [6,7]. Originally structured in five dimensions, the factor structure of the CD-RISC has revealed certain limitations in the multidimensional concept proposed. For that reason a new 10-item version was developed, which resulted in a stable scale with excellent psychometric properties [6].

As far as the authors know, the psychometric properties of the Spanish version of the 10-item CD-RISC have not been evaluated. Therefore, this study aimed to evaluate the validity and reliability of the Spanish version of the 10-item CD-RISC in young adults of Cuenca, Spain, in addition to verifying the single dimension factor structure.

## Findings

### Study design and population

Cross-sectional, observational study in which were invited to participate a total of 770 first-year university students, age 18 to 30 years, of the Castile-La Mancha University in Cuenca campus, Spain. Six hundred eighty-three (88.7%) students participated in the study and 681 correctly completed the questionnaires. Students who refused to participate in the study were similar in mean age and sex distribution to participants. We were unable to ask for the reason for their refusal due to compulsory indications in this sense of Clinical Research Ethics Committee.

The study protocol was approved by the Clinical Research Ethics Committee of Hospital Virgen de la Luz of Cuenca. All the subjects were asked to sign the informed consent to participate in the study after receiving oral and written information about the study objectives and procedures.

### Measurement variables and instruments

All the subjects were administered a battery of tests to determine, in addition to the sociodemographic variables:

#### - Resilience

This was evaluated using the 10-item CD-RISC [6,8], a self-administered questionnaire of 10 items designed as a Likert type additive scale with five response options (0 = never; 4 = almost always), which had a single dimension in the original version. The final score on the questionnaire was the sum of the responses obtained on each item (range 0-40) and the highest scores indicated the highest level of resilience. In order to define the final version, the version of the scale translated into Spanish provided by the authors of the original scale and was adapted with minimal changes [9].

#### - Mental health

The Mental Component Summary (MCS) of the SF-12 quality of life questionnaire, adapted and validated in Castilian Spanish was used [10].

#### -Quality of sleep

The Pittsburgh Sleep Quality Index (PSQI) [11] is a simple, short self-administered questionnaire, consisting of 19 questions for the patient and 5 more questions for the partner, and structured in seven dimensions. Each dimension was scored from 0 to 3 and the final score obtained was 0 to 21. The scale is negative and the highest score corresponds to the worst quality of sleep. Approved Spanish version was used [12].

### Questionnaire administration strategy

Students were convened for meetings in the classrooms of the respective centers, where the study objectives and procedures were explained. After the presentation, all the students who signed the informed consent were given the questionnaire to complete. Three investigators were in the classroom while students completed the questionnaires to avoid contamination between the responses of each one.

### Statistical analysis and validation process

#### Construct validity

Principal components analysis (PCA) was used to analyze a number of factors underlying the scale. The Bartlett sphericity test and KMO index were used to assess the suitability of the factor solution. An *eigenvalue *of 1 was used as a criterion for factor extraction. A sedimentation graph was used to analyze the suitability of the number of factors extracted.

The suitability of a single factor model underlying 10-item CD-RISC was analyzed by confirmatory factor analysis (CFA) with IBM SPSS Amos 19 software. Because of sex differences in resilience have been described [13], we tested if the factor structure of resilience construct was similar for both men and women, and analyses were performed for each sex and Chi-squared tests were used to examine differences in factors loadings between the sexes. As the sample sizes were relatively large (n = 681), the goodness of fit of the hypothetical models to the sample data was assessed with the Hu and Bentler criteria [14].

To test the factor structure of the 10-item CD-RISC, we splitted the sample into two subsamples randomly and conducted PCA and CFA using this two sub-samples respectively.

#### Convergent validity

The total 10-item CD-RISC score was categorized as: low resilience (first quartile), moderate resilience (second and third quartiles) and high resilience (fourth quartile). Given that the highest levels of resilience are associated with better mental health conditions [15] and that certain mental problems like anxiety and depression are associated with sleep disorders and less resilience [11,16], the convergent validity of the scale was analyzed, by gender, by ANCOVA models using MCS mean and PSQI mean as dependent variables, 10-item CD-RISC categories as fixed factors, and age as covariate. Effect sizes 'd' were calculated employing the estimated marginal means and were interpreted as small (0.20-0.50), moderate (0.51-0.80) or large (> 0.80) [17].

#### Reliability

The internal consistency of the scale was evaluated by calculating Cronbach's alpha coefficient. Test-retest reliability was examined in a subsample of 95 students selected randomly from all the sample subjects who completed this questionnaire two times, once when they were convened to participate in the study and the second time two weeks later. The tests-retest intraclass correlation coefficient was used in the reproducibility analysis of the 10-item CD-RISC.

Except for CFA, analyses were performed with IBM SPSS Statistics 19 software [18].

## Results

The final sample included 681 first-year university students, age range 18 to 30 years (mean = 20.08; SD = 4.12). Off these, 506 (73.86%) were women, percentage in accordance with the sex ratio in the University Campus of Cuenca, Spain. The mean score of 10-item CD-RISC for the total sample was 27.41 (SD = 6.36); students over 25 years showed scores significantly lower (27.06; SD = 6.36) than students under 25 years (29.86; SD = 6.52), p < 0.05. Also, the mean score of 10-item CD-RISC was significantly higher in men (29.47; SD = 5.80) than in women (26.46; SD = 6.43) p < 0.001.

None of the participants obtained a total score of 0 on the scale (floor effect), and only 2.3% of subjects obtained the maximum score (ceiling effect).

### Construct validity

**Figure 1:**
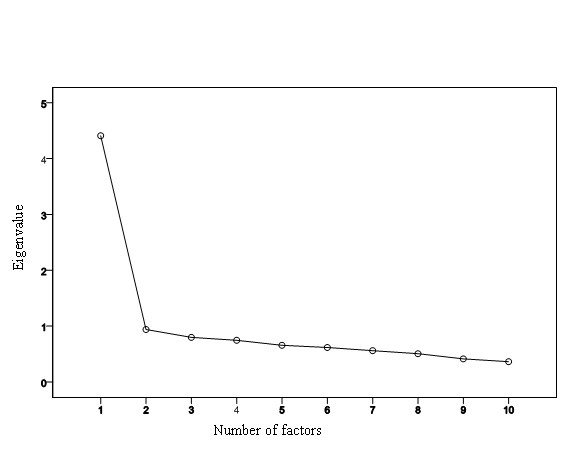
**Sedimentation graph of factor components of 10-item CD-RISC**.

The factor solution was adequate. The result of the KMO test was 0.90 and the Barlett sphericity was significant (χ^2 ^= 2074.7; gl = 45; p = 0.001). Only one factor showed an eigenvalue greater than 1. This factor explained 44.1% of the variance. The saturation of each item on the PCA is presented in Table 1. The sedimentation graph showed a single suitable factor solution (Figure 1).

**Table 1:** Factor structure of the 10-item CD-RISC.

Items	Saturation
1. Able to adapt to change	0.843
2. Can deal with whatever comes	0.834
3. Tries to see humorous side of problems	0.836
4. Coping with stress can strengthen me	0.838
5. Tends to bounce back after illness or hardship	0.849
6. Can achieve goals despite obstacles	0.833
7. Can stay focused under pressure	0.845
8. Not easily discouraged by failure	0.851
9. Thinks of self as strong person	0.828
10. Can handle unpleasant feelings	0.843

Cronbach α = 0.854

### Confirmatory factor analysis

**Figure 2:**
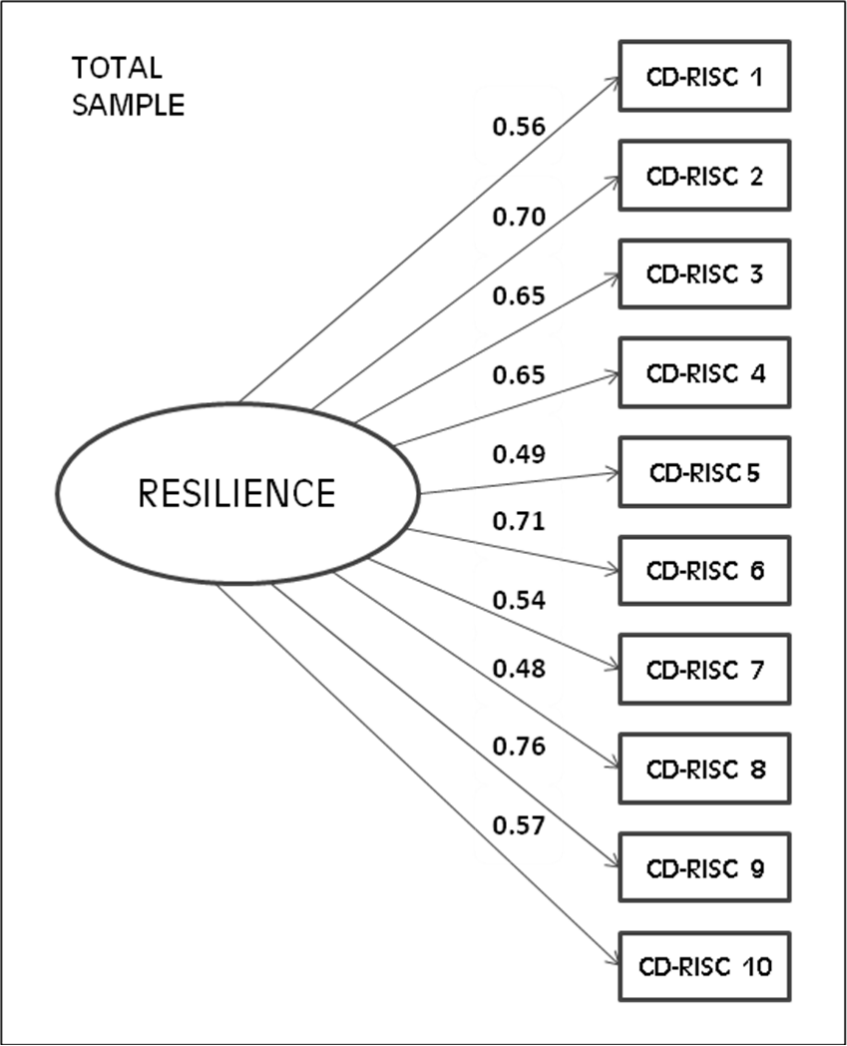
**Factor loading and goodness-of-fit indexes of one-factor model for the 10-items CD-RISC factor structure**. Total sample: n = 681; χ^2 ^= 159.4, df = 35, p = 0.001, CFI = 0.94 and SRMR = 0.041.

**Figure 3:**
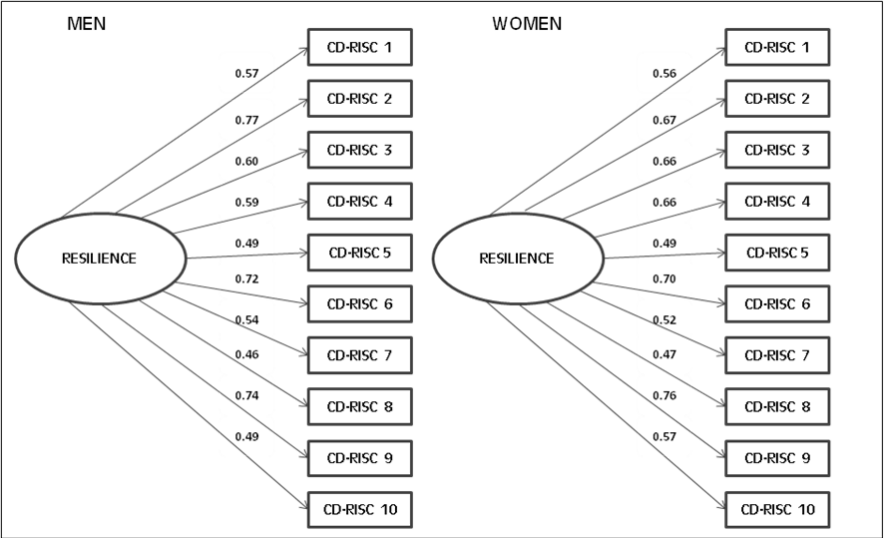
**Factor loading and goodness-of-fit indexes for our single-factor model for the CD-RISC-10 factor structure, by sex**. Men: n = 175; χ^2 ^= 135.8, df = 35, p = 0.001, CFI = 0.82 and SRMR = 0.073. Women: n = 506; χ^2 ^= 106.4, df = 35, p = 0.001, CFI = 0.95 and SRMR = 0.040.

(Figure 2: χ^2 ^= 159.4, df = 35, p = 0.001; CFI = 0.939; and SRMR = 0.041). The single factor model proposed for the CFA of the 10-item CD-RISC, by sex, is shown in Figure 3. The model displayed a good fit by sex and the factor loading showed no differences between men and women (Figure 3: χ^2^diff = 9.4, df = 9, p = 0.40).

We separated randomly the sample into two groups, and the PCA and CFA results did not show significant differences between groups.

### Convergent validity

The differences in the mean score of the MCS and in the mean score of the PSQI by resilience category, controlling for age, by gender, are shown in Table 2. The quality of sleep in both men and women was significantly worse in subjects with lower resilience scores. On the other hand, the score on the MCS was also significantly lower in both men and women in the lowest resilience category.

**Table 2:** Mean score of the SF-12 mental component and Pittsburgh sleep quality index by resilience category, controlling for age, by sex.

	PSQI Mean (SD)						
**10-item CD-RISC**	Low Resilience (Percentile <25)	Moderate Resilience (Percentile 25-75)	High Resilience (Percentile >75)	p	Effect size		

					1-2	1-3	2-3
					
Men	7.00 (3.31) n = 22	5.58 (2.56) n = 101	5.40 (3.16) n = 55	**0.029**	0.48	0.49	0.06
Women	6.99 (3.33) n = 167	6.22 (2.95) n = 245	5.86 (2.89) n = 90	**0.003**	0.24	0.36	0.12
Total	6.99 (3.32) n = 189	6.03 (2.85) n = 346	5.69 (2.99) n = 145	**<0.001**	0.31	0.41	0.12

	**MCS** **Mean (SD)**						

**10-item CD-RISC**	Low Resilience (Percentile <25)	Moderate Resilience (Percentile 25-75)	High Resilience (Percentile >75)	p	Effect size		

					1-2	1-3	2-3
					
Men	39.87 (6.15) n = 22	41.92 (5.07) n = 100	43.06 (5.78) n = 53	**0.013**	0.36	0.53	0.21
Women	37.37 (6.14) n = 166	38.89 (6.41) n = 240	42.00 (5.57) n = 89	**0.001**	0.24	0.79	0.52
Total	37.66 (6.18) n = 188	39.78 (6.20) n = 340	42.40 (5.65) n = 142	**<0.001**	0.34	0.80	0.44

### Reliability

The mean correlation between items was 0.37, the lowest value being 0.20 and the highest value being 0.57. The range of values of the item-total scale score correlation was 0.45 to 0.69. Cronbach's alpha was 0.854 and did not increase after eliminating any of the items. The intraclass correlation coefficient between the total score on the first 10-item CD-RISC questionnaire administered and the total score on the scale two weeks later was 0.711 (95%CI = 0.596-0.798); the Spearman correlation coefficient was 0.73 (Table 3).

**Table 3:** Correlation of the test-retest reliability analysis.

	Test	Test	Retest
N	681	95	95
Mean (SD)	27.41 (6.36)	27.03 (5.98)	27.74 (5.08)
Mean CD-RISC P_25_/_75_	23/32	22/31	24/32
Cronbach's alpha	0.854	0.831	0.807
Spearman correlation			0.732*

*p < 0.001

## Conclusions

The findings of our study confirmed that the Spanish version of the 10-item CD-RISC show good psychometric properties and a high level of reliability and validity in young adults. The findings also confirmed a single dimension underlying the 10 items of the scale.

The reliability of the Spanish version of the 10-item CD-RISC was similar to that of the original version (Cronbach's α of the original version = 0.85 and of the Spanish version = 0.85), and the weights in factor analysis were within the range of 0.48-0.76 on our scale and within the range of 0.44-0.74 in the original.

The factor structure of the CD-RISC is debated [7,19-21], and no consensus exists regarding the number of factors composing this scale. It has been observed [22] that eliminating the items that were highly correlated resulted in a unidimensional final 10-item scale that was easier to complete and provided essentially the same information as the 25-item version [6]. Our data confirm that a single factor underlies the resilience construct, as in the original 10-item CD-RISC version, and suggests that the 10-item CD-RISC is an unidimensional measure of resilience.

Sleep disturbances coincide frequently with mental and/or physical disorders [23,16]. Likewise, different studies made in adolescents have shown that subjects with a high level of resilience are less likely to present mental disorders, interpersonal conflicts, behavior disorders and poor academic performance [24,25]. A recent study has found that the variation in the 5HTTPR gene is associated with individual differences in resilience [26], which could predict the appearance of mental health problems. Our results support the convergent validity of the scale because they showed that both the quality of sleep and the mean score of the MCS worse in both men and women with lower resilience scores. Other studies have commonly used posttraumatic stress scales as measures of convergent validation [5,6]; in our study was not possible to use these kind of measures because of the low lifetime prevalence of severe stressful events at the age of our sample.

Nonetheless, these results should be interpreted with caution given the limitations of this study. As a cross-sectional study, the results do not establish predictive validity between the levels of resilience and the MCS and the PSQI. Moreover, the sample studied included only university student, so our results certainly cannot be extrapolated to the general population. However, among the strengths of our study it should be noted that this is the first validation study of the 10-item CD-RISC in Castilian Spanish, and that this short and simple instrument requires little time to complete and is thus efficiently administered. For that reason, it may be a suitable instrument for clinical use and in community studies.

## Competing interests

The authors declare that they have no competing interests.

## Authors' contributions

BNP conceived the study design and contributed to collect the data and to redact the manuscript. MSM conducted the statistical analysis. MDSP, RBG and JGC contributed to draft the manuscript. VMV is the main researcher of the study, and he contributed to conceive the study design, to conduce the statistical analysis and to redact the manuscript. All authors read and approved the final manuscript.
